# Salinity Stress Enhances the Antioxidant Capacity of *Bacillus* and *Planococcus* Species Isolated From Saline Lake Environment

**DOI:** 10.3389/fmicb.2020.561816

**Published:** 2020-09-14

**Authors:** Abdelrahim H. A. Hassan, Dalal Hussien M. Alkhalifah, Sulaiman A. Al Yousef, Gerrit T. S. Beemster, Ahmed S. M. Mousa, Wael N. Hozzein, Hamada AbdElgawad

**Affiliations:** ^1^Department of Food Hygiene and Control, Faculty of Veterinary Medicine, Beni-Suef University, Beni-Suef, Egypt; ^2^Department of Biology, College of Science, Princess Nourah Bint Abdulrahman University, Riyadh, Saudi Arabia; ^3^Department of Clinical Laboratory Science, College of Applied Medical Sciences, University of Hafr Al Batin, Hafr Al Batin, Saudi Arabia; ^4^Integrated Molecular Plant Physiology Research, Department of Biology, University of Antwerp, Antwerp, Belgium; ^5^Bioproducts Research Chair, Department of Zoology, College of Science, King Saud University, Riyadh, Saudi Arabia; ^6^Department of Botany and Microbiology, Faculty of Science, Beni-Suef University, Beni-Suef, Egypt

**Keywords:** salinity stress, *Bacillus*, *Planococcus*, stress markers, antioxidants

## Abstract

This study aims at exploiting salinity stress as an innovative, simple, and cheap method to enhance the production of antioxidant metabolites and enzymes from bacteria for potential application as functional additives to foods and pharmaceuticals. We investigated the physiological and biochemical responses of four bacterial isolates, which exhibited high tolerance to 20% NaCl (wt/vol), out of 27 bacterial strains isolated from Aushazia Lake, Qassim region, Saudi Arabia. The phylogenetic analysis of the 16S rRNA genes of these four isolates indicated that strains ST1 and ST2 belong to genus *Bacillus*, whereas strains ST3 and ST4 belong to genus *Planococcus*. Salinity stress differentially induced oxidative damage, where strains ST3 and ST4 showed increased lipid peroxidation, lipoxygenase, and xanthine oxidase levels. Consequently, high antioxidant contents were produced to control oxidative stress, particularly in ST3 and ST4. These two *Planococcus* strains showed increased glutathione cycle, phenols, flavonoids, antioxidant capacity, catalase, and/or superoxide dismutase (SOD). Interestingly, the production of glutathione by *Planococcus* strains was some thousand folds greater than by higher plants. On the other hand, the induction of antioxidants in ST1 and ST2 was restricted to phenols, flavonoids, peroxidase, glutaredoxin, and/or SOD. The hierarchical analysis also supported strain-specific responses. This is the first report that exploited salinity stress for promoting the production of antioxidants from bacterial isolates, which can be utilized as postbiotics for promising applications in foods and pharmaceuticals.

## Introduction

Oxidation of food can occur during various steps of handling and leads to the formation of bad flavors, loss of essential fatty acids, fat−soluble vitamins and other bioactive compounds, and development of possibly poisonous compounds, so turning fat−rich foods unsafe for human consumption (Scott, 1997; Min and Ahn, 2005). Additionally, lipids in living organisms, particularly polyunsaturated fatty acids in phospholipids of cell membranes, can also experience oxidation during regular aerobic metabolism or through exposure to other oxidizing agents (Halliwell, 1991; Beckman and Ames, 1998). Consequently, oxidative stress has an important role in the pathogenesis of many illnesses and health problems (Floyd and Hensley, 2002; Halliwell, 2006). Free radical–mediated oxidation of lipids is therefore an important concern for researchers and food processors (Shahidi and Zhong, 2010).

In this context, antioxidants have received considerable interest in recent years. Antioxidants are substances that when present at low concentrations compared to that of an oxidizable substrate delay or prevent oxidation by scavenging the free radicals (Halliwell, 1999). The addition of antioxidants has become the most efficient, appropriate, and economical strategy for protecting food lipids and accordingly avoiding deterioration of food quality. Also, in medicine, they are used as health-promoting agents owing to their capability to enhance the effectiveness of the body’s antioxidant defense mechanisms (Shahidi and Zhong, 2010). Commonly used antioxidants are either natural compounds such as tocopherols, phenols, polyphenols, carotenoids, ascorbic acid, erythorbic acid or their salts and derivatives, or synthetic compounds such as butylated hydroxyanisole (BHA), butylated hydroxytoluene, tert-butylhydroquinone (TBHQ), and propyl gallate. However, there has been a growing concern over the potential carcinogenic effects of synthetic antioxidants (Sherwin, 1990; Hirose et al., 1993). Therefore, BHA has been banned in food applications in Japan and some other countries. Similarly, TBHQ is forbidden in Canada, Japan, and the European Union. Hence, there is a global need to replace synthetic antioxidants with natural ones.

The study of natural sources of antioxidants revealed that numerous antioxidant compounds have been obtained from plants, mainly fruits, vegetables, nuts, and whole grains, as well as seafood, meat, and poultry (Shahidi and Zhong, 2010; Mohammadipanah and Momenilandi, 2018). Higher plants are considered the richest source of antioxidants and have been studied extensively (Kähkönen et al., 1999; Krishnaiah et al., 2011). Conversely, marine organisms such as algae and bacteria have recently received a rising concern from scientists in this field (Athukorala et al., 2003).

Bacteria are usually exposed to different challenging environmental stressors in their environments including unfavorable temperature, salinity, adverse pH, high osmolarity, radiations, and toxins (Aertsen and Michiels, 2004). To be able to survive with these stressors, bacteria either move to a favorable environment or adapt to changes. The bacterial response to compulsory stress is usually achieved by changes in the gene expressions of those genes whose products are essential to overcome the harmful nature of the stress (Hecker and Volker, 2001).

Salinity has become a major ecological and agronomic problem (Shabala et al., 2014). Accordingly, several pieces of research have studied the impact of salinity on eukaryotes, particularly plants (Parida and Das, 2005; Ambede et al., 2012; Kurum et al., 2013; Hussain et al., 2014; AbdElgawad et al., 2016; Abdelaal K.A. et al., 2020; Abdelaal K.A.A. et al., 2020; Osman et al., 2020), as well as prokaryotes such as cyanobacteria, and their mechanisms of tolerance to high salinity (Joset et al., 1996; Lu et al., 2006; Rao et al., 2007). These studies demonstrated that plants and algae respond to salt stress by inducing antioxidant metabolites and enzymes. Nevertheless, to the best of our knowledge, the data regarding the response of bacteria to salinity stress are largely absent.

Herein, we aimed at exploiting salinity stress as a simple and cheaper way to produce antioxidant metabolites from high salt-tolerant bacterial isolates. Therefore, we isolated 27 bacterial isolates from a marine environment represented by marine water and sediment samples collected from Aushazia Lake at Qassim region of Saudi Arabia. Four bacterial isolates showed high tolerance to 20% (wt/vol) salt stress and were named salt-tolerant strains (ST1–ST4) throughout the study. Based on their 16S rRNA sequence, ST1 and ST2 isolates were classified in the genus *Bacillus*, whereas ST3 and ST4 could represent new species of the genus *Planococcus*. Subsequently, we investigated the mechanism triggered by the bacterial cells of these four strains to resist the adverse salinity conditions in terms of their physiological and biochemical responses to salinity stress and their effect on the antioxidant metabolite production. We demonstrated that salinity promotes the production of antioxidant metabolites and enzymes, which can be utilized as postbiotics for potential application as functional additives to foods and pharmaceuticals to enhance food stability and to promote human health.

## Materials and Methods

### Bacterial Isolation and Growth Conditions Under High Salt Stress

Twenty-seven bacterial isolates were isolated from marine water and sediment samples collected from Aushazia Lake at Qassim region of Saudi Arabia (26°04′08.0″ N 44°09′45.1″ E). The isolation was carried out on M1 agar medium plates, which are composed of 10 g of starch, 4 g of yeast extract, 2 g of peptone, 18 g of agar, and 1 L of natural seawater, which had a salinity of approximately 3.2% of NaCl (Mincer et al., 2002). The isolation plates were incubated for 1 week at 30°C. The bacterial isolates were purified and preserved in sterile 20% glycerol at −80°C. Then, to perform the initial selection through testing the responses of these bacterial isolates to salinity stress, we grew them into the same isolation medium in a liquid form (M1 medium/seawater) but at different total salt concentrations: 3.2% NaCl wt/vol for control and 10, 15, and 20% NaCl wt/vol for salinity stress. Of 27 bacterial isolates, four were selected according to their ability to grow at the highest salt stress level, i.e., 20% NaCl wt/vol for further identification and experimentations. After incubation for 24 h in the same medium and incubation conditions, 1.5 mL of the bacterial culture and control medium were collected, and the absorbance was measured at 600 nm (OD_600_) to compare the growth under high salinity and control conditions. Then, initial inocula (10^5^ colony-forming units/mL) of the four selected strains were cultured in shake flasks at 180 revolutions/min (rpm) for 5 days in 250 mL of the same growth medium with 3.2% wt/vol NaCl for control and 20% wt/vol NaCl for salinity stress exposure. The pH of the control and treatment media was adjusted to 7.2, and the incubation temperature was 35°C, unless otherwise stated. The incubation period of the salinity stress experiment was set based on the preliminary results, which showed that the maximum induction of total antioxidant capacity (ferric-reducing/antioxidant power, FRAP) was achieved at the fifth day of incubation, and the plateau was reached by 4–5 days of incubation (Supplementary Figure S1). The optical densities (absorbance at 600 nm, OD_600 nm_) of the selected four ST isolate cultures grown under control or salinity stress (20% wt/vol NaCl) were estimated at 24 h of incubation (Supplementary Table S1) to verify culture growth before proceeding with the experiment.

### Bacterial Characterization and Identification by Phylogenetic Analysis

Four bacterial isolates were selected for further work based on their ability to grow at the highest salt stress (20% NaCl wt/vol), whereas all other isolates could not survive at that high salt concentration. For identification of the four isolates, the genomic DNA was extracted from the bacterial biomass using the DNeasy UltraClean Microbial Kit by Qiagen (Germany) following the manufacturer’s instructions. Polymerase chain reaction (PCR) amplification of the 16S rRNA gene was conducted using the universal primers (27F and 1492R) as previously described (Hozzein and Goodfellow, 2007). The sequencing of the PCR products was done by Macrogen, South Korea using the standard procedures. The obtained sequences were compared with available 16S rRNA gene sequences from the DDBJ, EMBL, and GenBank databases using the EzTaxon-e server (Kim et al., 2012). Multiple alignments with sequences of the related organisms were done using MEGA X (Kumar et al., 2018). Phylogenetic trees were generated using the neighbor-joining method (Saitou and Nei, 1987). The evolutionary distances were computed using the maximum composite likelihood method (Tamura et al., 2004), and they were in the units of the number of base substitutions per site. The resulting tree topologies were assessed by bootstrap analysis based on 1,000 resamplings (Felsenstein, 1985).

### Determination of Oxidative Stress Markers for Selected Isolates

At the end of the incubation period (5 days), separate cultures of each of the four strains, either grown under salinity stress or control condition, were transferred to 5 mL polypropylene tubes and centrifuged (10,000 × *g*, 10 min, 4°C). The resulting supernatant was filtered (0.22-μm pore size), and cell-free supernatants were collected for oxidative damage and antioxidant analyses. Malondialdehyde (MDA) content, an end product of lipid peroxidation, was assayed according to Hodges et al. (1999). Ten milligrams of freeze-dried bacterial cells were homogenized in 1 mL of 80% ethanol by using MagNA Lyser (Roche, Vilvoorde, Belgium; 7,000 rpm/1 min) and reacted with thiobarbituric acid (TBA) to produce pinkish-red chromogen TBA–MDA. Absorbance at 440, 532, and 600 nm was measured using a microplate reader (Synergy Mx; BioTek Instruments Inc., Vermont, VT, United States). MDA content was computed and expressed in nmol/g cell weight. Xanthine oxidase (EC 1.1.3.22) was measured based on xanthine/xanthine oxidase system of O_2_^–^ generation given by Beauchamp and Fridovich (1971). Xanthine oxidase activity was assessed by the reduction of XTT (2,3-bis(2-methoxy-4-nitro-5-sulfophenyl)-2H-tetrazolium-5-carboxanilide sodium salt) in the absence (blank) and the presence of xanthine at 470 nm. Lipoxygenase (EC 1.13.11.12) activity was analyzed according to Axelrod et al. (1981) and measured spectrophotometrically at 234 nm in a Shimadzu UV-160 spectrophotometer (Shimadzu Corporation, Kyoto, Japan).

### Determination of the Overall Antioxidant Capacity

Total antioxidant capacity FRAP assay was done by grinding 30 mg freeze-dried bacterial cells in liquid nitrogen and extracted in 2 mL of ice-cold 80% ethanol. FRAP reagent (0.3 M acetate buffer (pH 3.6), 0.01 mM 2,4,6-Tris(2-pyridyl)-s-triazine (TPTZ) in 0.04 mM HCl and 0.02 M FeCl_3_⋅6H_2_O was mixed with the extract for 30 min and measured at 600 nm using a microplate reader (Benzie and Strain, 1999). 6-hydroxy-2,5,7,8-tetramethylchromane-2-carboxylic acid (Trolox) was used as standard. The 1,1-diphenyl-2-picrylhydrazyl (DPPH) free radical–scavenging activity was also estimated in 30 mg freeze-dried bacterial cells according to the method of Cheung et al. (2003). Additionally, the superoxide-scavenging (SOS) activity was also measured in 30 mg freeze-dried bacterial cells as described by Srinivasan et al. (2007).

### Determination of the Antioxidant Metabolites

Total polyphenols and flavonoids were extracted from 30 mg of freeze-dried bacterial cells in 80% ethanol (vol/vol) and estimated according to Zhang et al. (2006) and Chang et al. (2002). Gallic acid and quercetin were used as standards, respectively. Individual phenolic acids and flavonoids were determined by using the method described by Hamad et al. (2015). Approximately 50 mg of freeze-dried bacterial cells were homogenized in an acetone–water solution (4:1 vol/vol) for 24 h. Phenolic acids and flavonoids were quantified on a Shimadzu high-performance liquid chromatography (HPLC) system (SCL-10 AVP, Japan), equipped with a Lichrosorb Si-60, 7 μm, 3 × 150-mm column and a diode array detector (SPDM10AVP). The mobile phase water–formic acid (90:10, vol/vol) and acetonitrile–water–formic acid (85:10:5, vol/vol/vol) were employed at a flow rate of 0.8 mL min^–1^. The extract was filtered and centrifuged, and the resulting supernatant was evaporated under vacuum; next, the residue was resuspended in HPLC–grade methanol. Baicalein (100 μg/mL) was used as an internal standard, and the concentration of phenolic compounds was determined based on the corresponding standard. Glutathione (GSH) was also obtained by HPLC using the method of Potters et al. (2004). The redox status (GSH/tGSH) was calculated as the ratio of the reduced form to the total concentration of the antioxidant (El-Soud et al., 2013; AbdElgawad et al., 2015, Casasole et al., 2017). All antioxidant metabolites assay analyses were performed in at least three biological replicates.

### Determination of Enzymatic Antioxidant Mechanism

Enzyme activities were assessed in a semi–high-throughput setup (AbdElgawad et al., 2016; Avramova et al., 2017, Melandri et al., 2020). Superoxide dismutase (SOD) activity was determined by measuring the inhibition of nitro-blue tetrazolium reduction (ε550 = 12.8 mM^–1^ cm^–1^) (Dhindsa et al., 1982). Peroxidase (POX) activity was measured by the oxidation of pyrogallol in 100 mM phosphate buffer (ε430 = 2.46 mM^–1^ cm^–1^) (Kumar and Khan, 1983). Catalase (CAT) activity was estimated by monitoring the H_2_O_2_ decomposition at 240 nm (ε240 = 39.4 M^–1^ cm^–1^) (Aebi, 1984). GSH reductase (GR) activity was measured by monitoring the decline in NADPH (ε340 = 6.22 mM^–1^ cm^–1^) according to Murshed et al. (2008). It was assayed in 50 mM HEPES, pH 8. GSH *S*-transferase (GST) activity was assayed by measuring the conjugation of GSH to 1-chloro-2,4-dinitrobenzene at 340 nm (Habig et al., 1974). Glutaredoxin (Grx) activity was determined by measuring the reduction of 2-hydroxy-ethyl-disulfide by GSH in the presence of NADPH and yeast GR as described by Lundberg et al. (2001). GSH POX (GPX) activity was monitored as reported by Drotar et al. (1985), in a coupled enzyme assay with GR. Thioredoxin (TRD) activity was assayed by calculating NADPH oxidation at 340 nm (Wolosiuk et al., 1979).

### Statistical Analysis

The statistical analysis was carried out using the SPSS statistical package (SPSS Inc., Chicago, IL, United States). Each experiment was replicated at least three times. One-way analysis of variance was done. Tukey test was used as the *post hoc* test for the separation of means (*P* < 0.05).

## Results

### Bacterial Isolation and Growth Conditions Under High Salt Stress

A total of 27 bacterial isolates were retrieved from the collected samples; four strains of them exhibited a good growth capability under the highest salt concentration (20% NaCl), whereas the remaining 23 isolates could not survive at that high salt stress. Consequently, the four salt-tolerant strains (ST1, ST2, ST3, and ST4) were selected for further investigations.

### Bacterial Characterization and Identification

**FIGURE 1:**
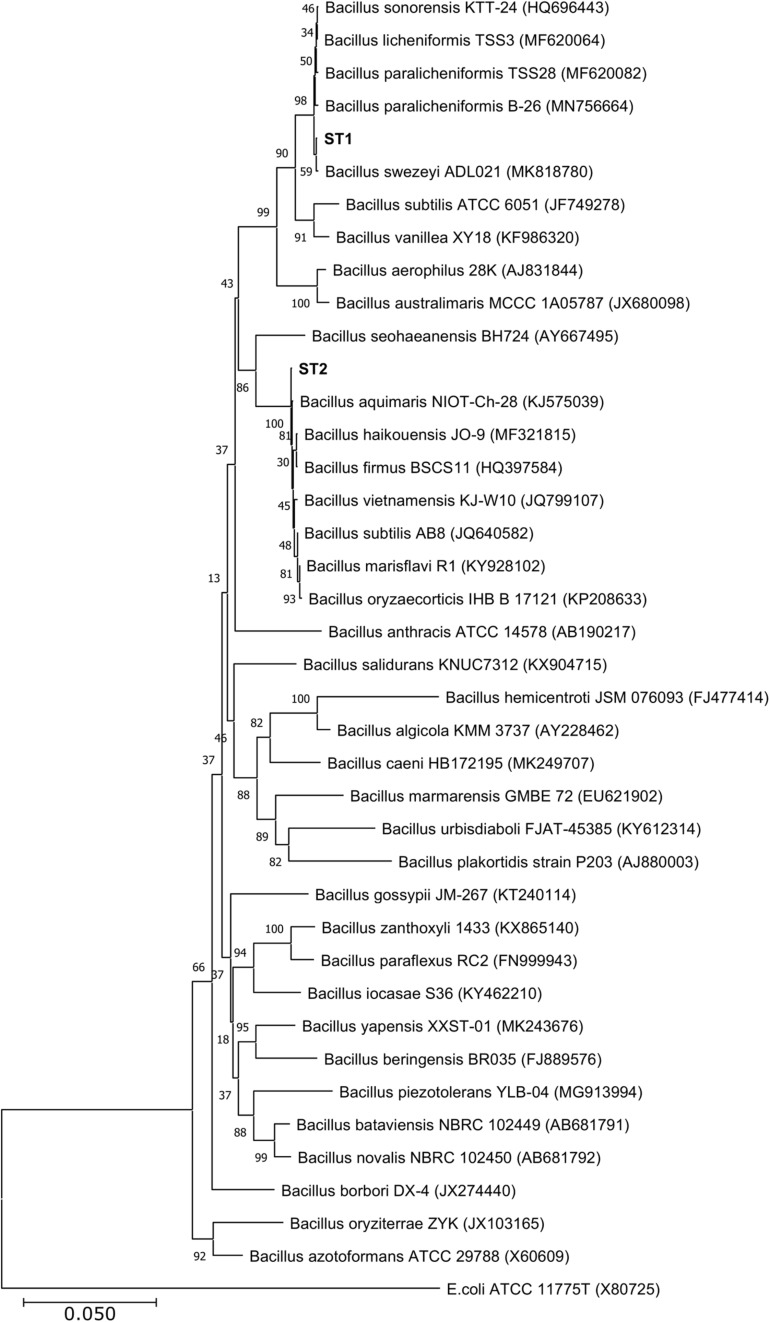
Neighbor-joining phylogenetic tree showing the relationships between the salt-tolerant *Bacillus* strains from the present study (ST1 and ST2) and the closely related species. All ambiguous positions were removed for each sequence pair (pairwise deletion option). *Escherichia coli* ATCC 11775 was used as the out–group.

**FIGURE 2:**
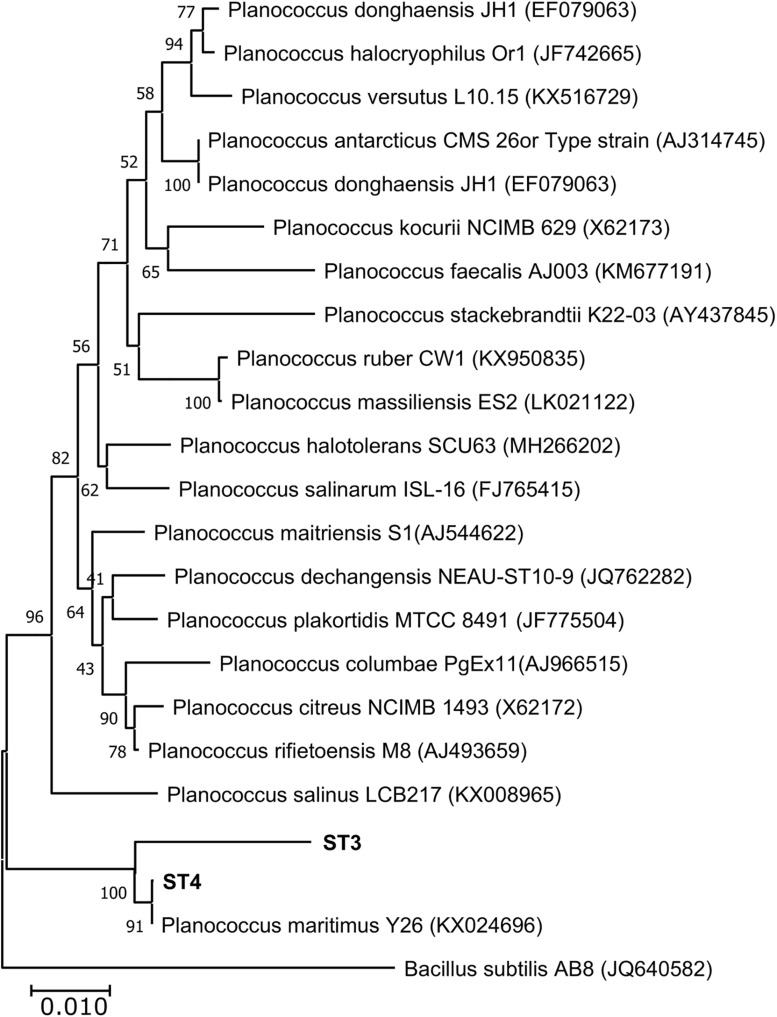
Neighbor-joining phylogenetic tree showing the relationships between the salt-tolerant *Planococcus* strains from the present study (ST3 and ST4) and the closely related species. All ambiguous positions were removed for each sequence pair (pairwise deletion option). *Bacillus subtilis* AB8 was used as the out–group.

Of the four isolates, two isolates (ST1 and ST2) were rod-shaped, and the other two (ST3 and ST4) were sphere-shaped. Comparison of the16S rRNA sequences of the four strains with the available prokaryotic sequences in the public databases indicated that ST1 and ST2 belong to genus *Bacillus* (Figure 1), whereas the strains ST3 and ST4 belong to genus *Planococcus*. The phylogenetic analysis revealed that ST1 is closely related to *Bacillus swezeyi*, with a similarity percentage of 99.23%, and ST2 is closely related to *Bacillus aquimaris* with a similarity percentage of 99.37% (Figure 1). On the other hand, ST4 was closely related to *Planococcus maritimus* with a similarity percentage of 99.51%, whereas strain ST3 could represent a new species of the genus *Planococcus* with the highest similarity percent of 96.33% (Figure 2). The obtained sequences of the four strains were submitted to the National Center for Biotechnology Information, and the following accession numbers: MT704984, MT704985, MT704986, and MT704987 were provided for ST1, ST2, ST3, and ST4, respectively.

### Oxidative Damage Markers Under Salinity Stress

**FIGURE 3:**
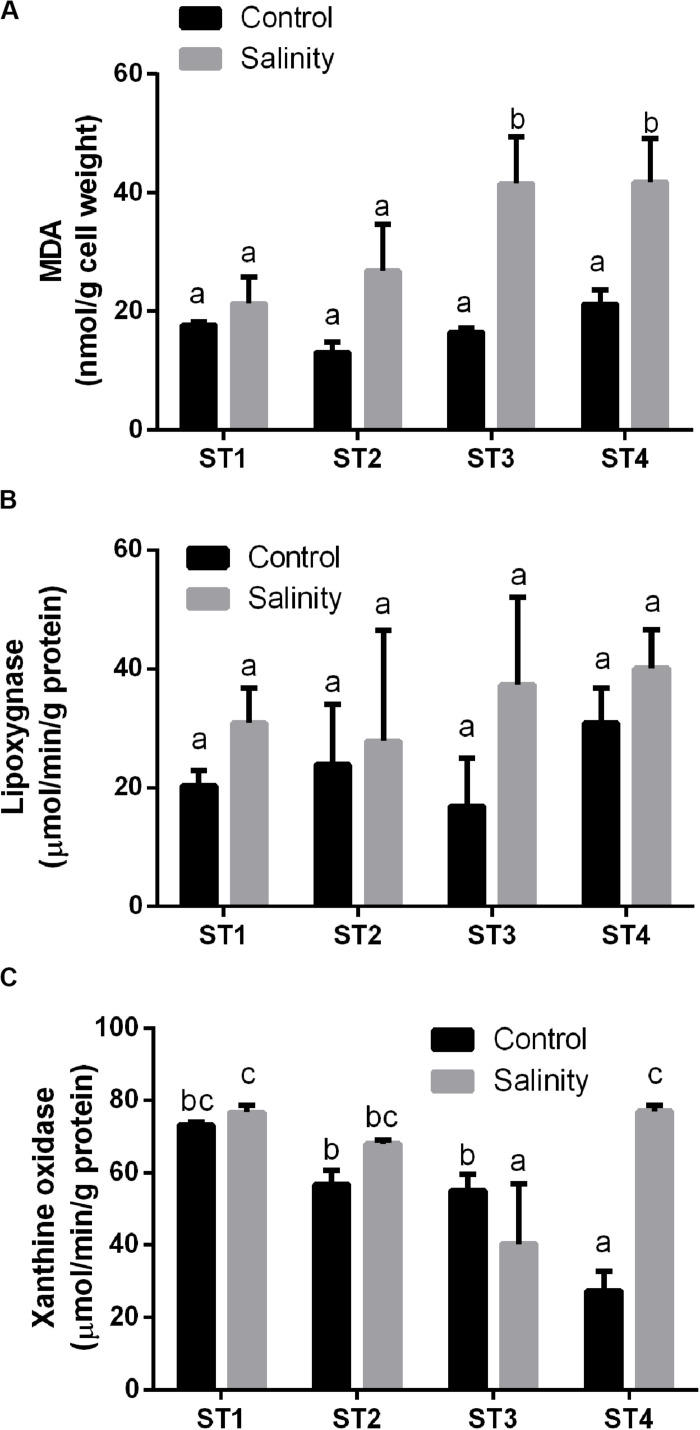
The oxidative damage markers of four salt-tolerant bacterial isolates (ST1, ST2, ST3, and ST4) in terms of **(A)** lipid peroxidation (MDA), **(B)** lipoxygenase activity, and **(C)** xanthine oxidase activity under control and salinity stress conditions. Data are represented by the mean of at least three replicates, and error bars represent the standard error. Different small letters (a, b, c…) above bars indicate significant differences between means at *p* < 0.05.

We determined the oxidative stress that was induced in the four salt-tolerant bacterial isolates by salinity stress. Lipid peroxidation (MDA), lipoxygenase, and xanthine oxidase of those four bacterial strains were compared under control condition and salinity stress (Figure 3). MDA levels of all isolates were less than 22 nmol/g cell weight under control conditions. In response to 20% (wt/vol) NaCl, MDA levels increased in all lines, but this increase was slight and not significant in ST1 and ST2, whereas the levels nearly doubled in ST3 and ST4 isolates to about 41 nmol/g cell weight (*p* < 0.05; Figure 3A). Compared to non-stressed strains, salinity stress also increased the activity of the oxidative marker lipoxygenase in all four strains; however, for individual strains, the difference was not significant (Figure 3B). Exposure to salinity did not induce significant differences in xanthine oxidase activity in isolates ST1 and ST2, whereas ST3 isolate showed a significant reduction by salinity exposure, and ST4 showed a significant elevation (*p* < 0.05; Figure 3C).

### Antioxidant Defense System

#### Salt Stress Improved the Overall Antioxidant Capacity of Salt-Tolerant Isolates

**FIGURE 4:**
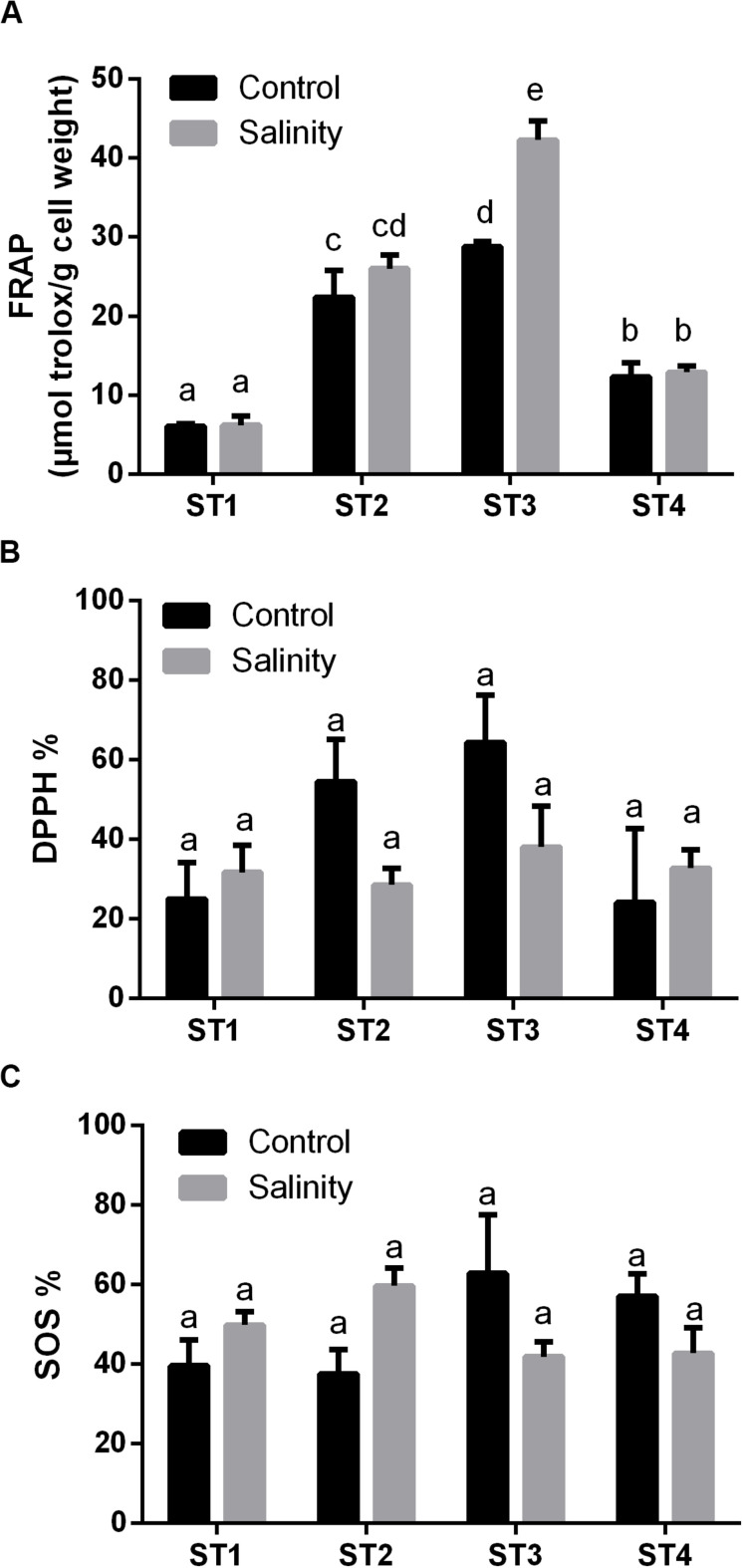
The overall antioxidant capacity of four salt-tolerant bacterial isolates (ST1, ST2, ST3, and ST4) in terms of **(A)** FRAP, **(B)** DPPH %, and **(C)** superoxide scavenging (SOS) under control and salinity stress conditions. Data are represented by the mean of at least three replicates, and error bars represent the standard error. Different small letters (a, b, c…) above bars indicate significant differences between means at *p* < 0.05.

Next, we investigated the overall antioxidant levels and the effects of salinity-stress on the overall antioxidant capacity, namely, FRAP, 2,2−diphenyl−1−picrylhydrazyl (DPPH) and SOS of the four salt-tolerant isolated strains (Figure 4). Generally, ST3 followed by ST2 exhibited the highest FRAP activity under control condition and salinity stress led to a further increase in these lines (*p* < 0.05; Figure 4A). On the other hand, in ST1 and ST4, FRAP activities were low in both control and stressed samples, and salinity stress had no effect on these lines (Figure 4A). Similar to FRAP, the highest DPPH % was found in ST3 followed by ST2 under control conditions. However, exposure to salinity reduced DPPH % in those lines by about 50% (Figure 4B). On the contrary, DPPH activity was low and unaffected by salinity in ST1 and ST4. SOS activity, an indicator for total antioxidant capacity, tended to be the highest in ST3 and ST4 and decreased in response to salt, whereas these levels increased in ST1 and ST2, albeit not significantly (Figure 4C). Thus, we found no correlation between oxidative damage and FRAP or DPPH, whereas the lines that showed least oxidative damage (ST1 and ST2) upregulated the SOS levels, in contrast to ST3 and ST4 where these levels were downregulated.

#### Salt Stress Increased the Antioxidant Metabolites in Salt-Tolerant Isolates

**FIGURE 5:**
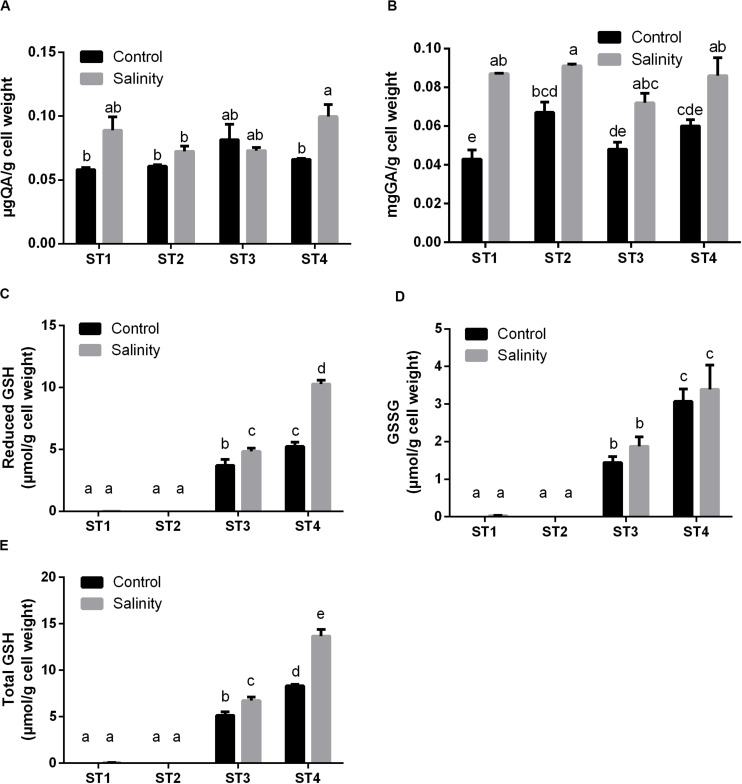
The antioxidant metabolites of four salt-tolerant bacterial isolates (ST1, ST2, ST3, and ST4) in terms of **(A)** flavonoids, **(B)** polyphenols, **(C)** reduced GSH, **(D)** oxidized GSH (GSSG), and **(E)** total GSH per gram of bacterial cell weight under control and salinity stress conditions. Data are represented by the mean of at least three replicates, and error bars represent the standard error. Different small letters (a, b, c…) above bars indicate significant differences between means at *p* < 0.05.

Following, we measured the production of polyphenols and flavonoids by isolated bacterial strains after salinity exposure. Under control conditions, there were no significant differences in the levels of flavonoids between the isolates. Flavonoids levels were increased by salinity in ST1, ST2, and ST4, although this elevation was significant only in ST4 (*p* < 0.05; Figure 5A). Total phenol levels under control condition were highest in ST2 followed by ST4, ST3, and finally ST1. Exposure to salinity stress significantly increased these levels in all isolates (*p* < 0.05; Figure 5B).

Then, we assessed individual phenolic acids and flavonoids under control and salinity stress conditions (Table 1). We noticed that ferulic acid, protocatechuic acid, and syringic acid were the most predominant phenolic acids, and salinity stress significantly (*p* < 0.05) induced them in almost all isolates, whereas isoquercitrin was the most prevalent flavonoid and salinity stress significantly (*p* < 0.05) boosted it in almost all isolates, as well. Additionally, salinity stress aided in increasing most of the measured phenolic acids and flavonoids in the four ST strains and that elevations were significant (*p* < 0.05) on many occasions. On the other hand, sinapic acid, pyrogallol, isorhamnetin, and kaempferol were undetectable in ST1 and ST2 (*Bacillus* strains) at both control and stress conditions. Also, taxifolin was undetectable in ST3 and ST4 (*Planococcus* strains) at both control and stress conditions (Table 1).

**TABLE 1:** Individual phenolic acids and flavonoids produced by the four salt-tolerant isolates (ST1, ST2, ST3, and ST4) under control or salinity stress conditions.

Phenolic acids	ST1		ST2		ST3		ST4	
	Control	Salinity	Control	Salinity	Control	Salinity	Control	Salinity
Caffeic acid	0.16 ± 0.028^bc^	0.27 ± 0.011^a^	0.09 ± 0.002^de^	0.20 ± 0.005^b^	0.08 ± 0.003^e^	0.18 ± 0.007^bc^	0.06 ± 0.003^e^	0.14 ± 0.006^cd^
Ferulic acid	0.84 ± 0.036^c^	2.14 ± 0.305^ab^	0.64 ± 0.027^c^	2.43 ± 0.346^a^	1.04 ± 0.044^bc^	2.81 ± 0.401^a^	0.99 ± 0.085^c^	ND
Protocatechuic acid	1.22 ± 0.105^ef^	2.58 ± 0.221^bc^	0.97 ± 0.168^f^	2.93 ± 0.073^ab^	2.01 ± 0.347^cde^	3.78 ± 0.095^a^	1.40 ± 0.074^def^	2.15 ± 0.114^bcd^
Sinapic acid	0.10 ± 0.004^b^	ND	ND	ND	0.15 ± 0.006^b^	0.52 ± 0.074^a^	0.21 ± 0.009^b^	0.20 ± 0.028^b^
Chlorogenic acid	0.53 ± 0.046^e^	1.19 ± 0.102^bc^	0.39 ± 0.01^e^	1.30 ± 0.032^b^	0.68 ± 0.017^de^	0.46 ± 0.012^e^	0.91 ± 0.048^cd^	2.55 ± 0.135^a^
Syringic acid	0.44 ± 0.019^e^	0.98 ± 0.041^d^	0.37 ± 0.016^e^	0.42 ± 0.018^e^	1.69 ± 0.071^c^	4.44 ± 0.188^a^	1.97 ± 0.084^c^	3.87 ± 0.164^b^
Pyrogallol	0.57 ± 0.024^a^	ND	ND	ND	0.09 ± 0.004^d^	0.25 ± 0.011^c^	0.12 ± 0.003^d^	0.38 ± 0.054^b^
Gallic acid	0.10 ± 0.004^e^	0.23 ± 0.01^cd^	0.09 ± 0.004^e^	0.3 ± 0.013^c^	0.15 ± 0.004^de^	0.43 ± 0.011^b^	0.22 ± 0.009^cd^	1.48 ± 0.062^a^
Flavonoids								
Catechin	0.39 ± 0.017^c^	0.83 ± 0.035^a^	0.36 ± 0.064^cd^	0.65 ± 0.028^b^	0.25 ± 0.010^de^	0.54 ± 0.023^b^	0.20 ± 0.008^e^	0.14 ± 0.006^e^
Resorcinol	0.81 ± 0.034^ab^	0.78 ± 0.033^abc^	0.68 ± 0.029^bc^	0.98 ± 0.041^a^	0.12 ± 0.005^e^	0.43 ± 0.061^d^	0.17 ± 0.007^e^	0.58 ± 0.083^cd^
Quercetin	0.63 ± 0.026^a^	0.50 ± 0.021^b^	ND	ND	0.15 ± 0.006^c^	0.44 ± 0.019^b^	0.21 ± 0.009^c^	ND
Quercetrin	0.13 ± 0.011^f^	0.39 ± 0.033^cd^	0.97 ± 0.024^a^	0.40 ± 0.010^cd^	0.23 ± 0.039^ef^	0.54 ± 0.023^c^	0.32 ± 0.057^de^	0.73 ± 0.031^b^
Luteolin	0.05 ± 0.008^b^	0.03 ± 0.005^b^	0.99 ± 0.042^a^	0.94 ± 0.133^a^	0.06 ± 0.011^b^	0.18 ± 0.025^b^	0.08 ± 0.003^b^	0.06 ± 0.009^b^
Apigenin	0.24 ± 0.010^de^	0.16 ± 0.007^e^	0.2 ± 0.008^e^	0.67 ± 0.028^b^	0.35 ± 0.015^d^	1.04 ± 0.044^a^	0.51 ± 0.021^c^	1.11 ± 0.047^a^
Isoquercitrin	0.90 ± 0.038^bcd^	1.05 ± 0.044^bc^	0.72 ± 0.031^d^	1.85 ± 0.078^a^	0.8 ± 0.034^cd^	1.86 ± 0.079^a^	0.81 ± 0.069^cd^	1.18 ± 0.101^b^
Rutin	0.07 ± 0.003^e^	0.16 ± 0.007^cd^	0.07 ± 0.013^e^	0.05 ± 0.002^e^	0.1 ± 0.002^de^	0.29 ± 0.007^b^	0.18 ± 0.032^c^	0.42 ± 0.018^a^
Isorhamnetin	ND	ND	ND	ND	0.1 ± 0.004^c^	0.30 ± 0.013^b^	0.14 ± 0.003^c^	0.39 ± 0.010^a^
Taxifolin	0.32 ± 0.014^b^	0.72 ± 0.03^a^	0.34 ± 0.059^b^	0.24 ± 0.010^b^	ND	ND	ND	ND
Kaempferol	ND	ND	ND	ND	0.45 ± 0.019^c^	1.32 ± 0.056^b^	0.64 ± 0.027^c^	1.79 ± 0.076^a^
Vanillin	0.08 ± 0.003^d^	0.91 ± 0.039^ab^	0.67 ± 0.028^bc^	1.14 ± 0.163^a^	0.12 ± 0.005^d^	0.42 ± 0.060^cd^	0.17 ± 0.007^d^	0.57 ± 0.081^bc^

*Data are represented by means of at least three replicates ± standard errors. ND, not detectable. Different small letter superscripts (a, b, c…) within row indicate significant differences between means at p < 0.05.*

Additionally, we determined the levels of GSH including the reduced GSH, oxidized GSH (GSSG), and total GSH form. In general, salinity exposure enhanced the production of these metabolites. Apparently, ST1 and ST2 isolates contained negligible levels of all forms of GSH under control and salinity stress conditions. On the other hand, GSH and GSSG levels in ST3 and ST4 isolates were already higher under control conditions, and salinity further increased these levels, albeit only significantly for GSH (*p* < 0.05, Figures 5C–E). Thus, the lines that had the most oxidative damage (ST3 and ST4) paradoxically appeared to contain much higher levels of GSH.

#### Salt Stress Enhanced the Enzymatic Antioxidant Activities of Salt-Tolerant Isolates

**FIGURE 6:**
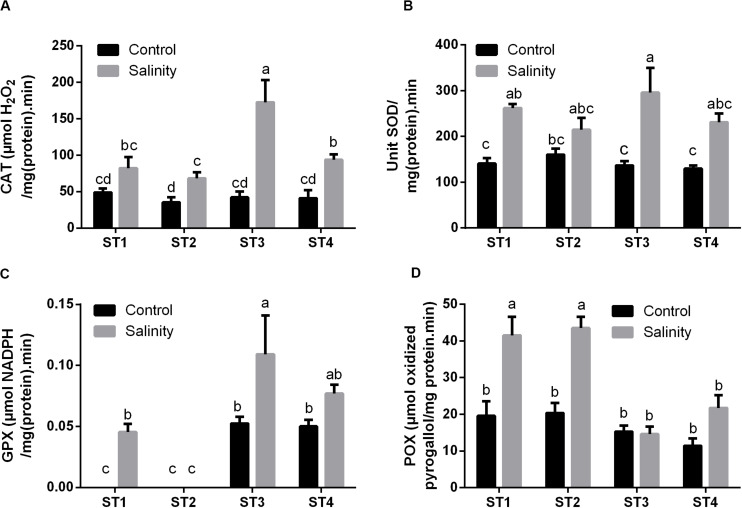
The antioxidant enzyme activities that directly scavenge ROS of four salt-tolerant bacterial isolates (ST1, ST2, ST3, and ST4) including **(A)** CAT, **(B)** SOD, **(C)** GPX, and **(D)** POX under control and salinity stress conditions. Data are represented by the mean of at least three replicates, and error bars represent the standard error. Different small letters (a, b, c…) above bars indicate significant differences between means at *p* < 0.05.

Next to antioxidant metabolites, reactive oxygen species (ROS) can be neutralized by a number of antioxidant enzymes. Therefore, we then measured the activity of common antioxidant enzymes that directly scavenge ROS: CAT, SOD, GPX, and POX in the selected strains under control and salinity-induced stress conditions. CAT activity was quite similar in ST1, ST2, ST3, and ST4 at control condition [48.90, 35.26, 42.26, and 40.92 μmol H_2_O_2_/mg (protein)⋅min, respectively], whereas by exposing them to salinity, it was increased in the four isolates, and this elevation was significant (*p* < 0.05) in ST2, ST3, and ST4 (Figure 6A). Likewise, SOD activity under control conditions was similar in the four isolates, whereas salinity increased this in all isolates, which was significant in ST1 and ST3 (*p* < 0.05; Figure 6B). As ST1 and ST2 did not have GSH as mentioned in Figure 5, we could not detect GPX in them under control conditions; interestingly, however, this capacity was significantly elevated by exposure to salinity in the case of ST1 (*p* < 0.05). In ST3 and ST4, GPX enzyme activity was increased by salinity exposure, albeit only significantly in ST3 (*p* < 0.05; Figure 6C). ST1 and ST2 showed the highest POX activities under control conditions. Salinity exposure doubled the POX activity in these lines (*p* < 0.05). In ST3 and ST4, POX activities were lower under control conditions, and the upregulation was absent or not significant (Figure 6D).

**FIGURE 7:**
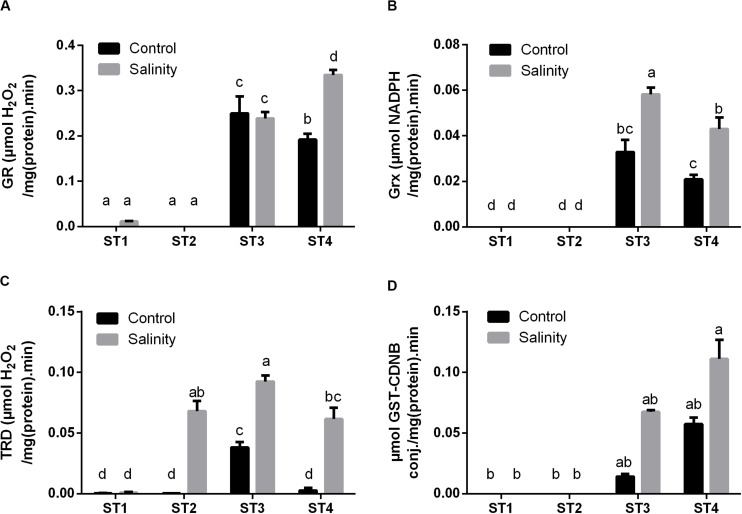
The antioxidant enzyme activities that have reducing activities to the antioxidant metabolites of four salt-tolerant bacterial isolates (ST1, ST2, ST3, and ST4) including **(A)** GR, **(B)** Grx, and **(C)** TRD, as well as the detoxification enzyme **(D)** GST activity under control and salinity stress conditions. Data are represented by the mean of at least three replicates, and error bars represent the standard error. Different small letters (a, b, c…) above bars indicate significant differences between means at *p* < 0.05.

Additionally, we have investigated the enzyme activities, which produce the reduced active form of several antioxidant metabolites, i.e., GR, Grx, and TRD. Apparently, ST1 and ST2 isolates showed no or negligible activity of the GR enzyme at both control and salinity stress conditions (Figure 7A). While GR activity in ST3 and ST4 was noticeable under control conditions, then by exposure to salinity stress, it was significantly increased in ST4 (*p* < 0.05), whereas there was no significant change in the case of ST3. Isolates ST1 and ST2 did not produce Grx under control conditions or by exposure to salinity stress (Figure 7B). On the other hand, the Grx activity of ST3 and ST4 was significantly (*p* < 0.05) duplicated after exposure to salinity stress. In Figure 7C, seemingly, TRD activities in ST1, ST2, and ST4 at control conditions were close to zero [μM H_2_O_2_/mg (protein)⋅min], whereas in ST3, it was significantly higher than other isolates, close to 0.05 μM H_2_O_2_/mg (protein)⋅min. Under salinity-induced stress, ST1 showed no change, whereas TRD activities were significantly increased in the other three isolates (*p* < 0.05).

Moreover, GST activity, as an important detoxification enzyme, was measured in the selected strains under both control and salinity stress condition (Figure 7D). Interestingly, the obtained results showed that ST1 and ST2 have no GST activity at both control and salinity conditions, whereas ST3 and ST4 exhibited high GST activity under control conditions, which were further increased by salinity stress (*p* < 0.05).

### Strain-Specific Responses

**FIGURE 8:**
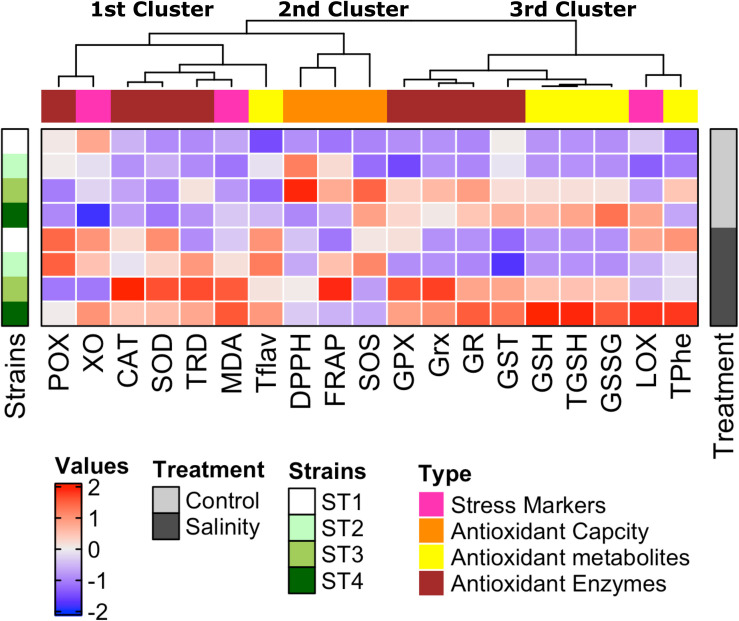
Strain-specific responses of four salt-tolerant bacterial isolates (ST1, ST2, ST3, and ST4) to the stress induced by high salt concentration at both physiological and biochemical levels shown by the hierarchical graph. The measured parameters represented by damage markers, overall antioxidant capacity, antioxidant metabolites, and antioxidant enzymes are grouped into three main clusters based on their response to high salt stress.

To give an overview of the differential biochemical changes induced by salinity stress in target strains, hierarchical clustering analysis of all results was generated using multi-experimental viewer (MEV) software. The analysis of the hierarchical graph (Figure 8) suggests strain-specific responses to the stress induced by the high salt concentration at both physiological and biochemical levels. The measured parameters represented by damage markers, overall antioxidant capacity, antioxidant metabolites, and antioxidant enzymes are grouped into three main clusters based on their responses. The first cluster consists of oxidative stress markers (xanthine oxidase and MDA), an antioxidant metabolite (total flavonoids), and antioxidant enzymes (POX, CAT, SOD, and TRD), which were improved mostly in all salinity-stressed isolates in comparison with control. The second cluster consists of antioxidant capacity markers (FRAP, DPPH, and SOS), which were clearly higher in ST2 and ST3 isolates under both control and salinity stress conditions than the other two isolates. The third cluster is composed of totals phenols, GSH forms (reduced, oxidized, and total GSH), stress marker (lipoxygenase), and antioxidant enzymes (GPX, Grx, GR, and GST), which were significantly greater in isolates ST3 and ST4 than ST1 and ST2, as well as salinity stress induced a significant enhancement in these measurements in ST3 and ST4.

## Discussion

It has always been known that most mechanisms that control the health-promoting applications of beneficial bacteria require bacteria to be viable such as in probiotics. Yet, a new term, *postbiotic*, has come out to signify that dead bacterial cells, bacterial portions, or cell lysates may also present functional advantages to the body by offering extra bioactivities (Aguilar-Toalá et al., 2018). Postbiotics, also known as metabiotic, biogenics, or simply metabolites, refers to soluble compounds secreted by live bacteria or liberated after bacterial lysis, such as antioxidant enzymes, antioxidant metabolites, polysaccharides, peptides, peptidoglycan-derived muropeptides, and organic acids. Postbiotics have attracted the interest of many researchers, given their simple chemical structure, high safety, long shelf life, and their content of numerous signaling molecules, which offer several functional properties such as anti-inflammatory, immunomodulatory, antihypertensive, antiproliferative, and antioxidant activities (Konstantinov et al., 2013; Cicenia et al., 2014). Accordingly, enhancing postbiotic production from bacteria could be of great significance in this field.

Reactive oxygen species production is one main signal to stimulate induced-antioxidant capacity in plants or microorganisms. The production of ROS by higher plants and algae is stimulated by various environmental stresses, for instance, exposure to high light levels (Foyer et al., 1997), drought (Melandri et al., 2020), heavy metals (Okamoto et al., 2001; Hamed et al., 2017, 2019), high salt concentrations (Meneguzzo et al., 1999; Zhang et al., 2013), high temperature (Rao et al., 1995), UV irradiation (Malanga and Puntarulo, 1995), air pollutants including ozone (Sharma et al., 1996), water stress (Boo and Jung, 1999), and herbicides (Tanaka, 1994). Accordingly, plant and algal cells exploit their defensive antioxidant mechanisms to tackle the threats posed by the generation of ROS. The antioxidant mechanisms include enzymatic and non-enzymatic defense systems (Okamoto et al., 2001). Hence, we expect similar responses of bacterial cells to environmental stress factors.

Of these environmental stressors, salinity has raised as a severe issue affecting agricultural productivity, as well as the persistence and distribution of wild plants (Shabala et al., 2014). Salinity induces a variety of responses in plants and algae including morphological, physiological, biochemical, and molecular changes. It causes ionic imbalance that results in toxicity, osmotic stress, and generation of ROS (Ambede et al., 2012; Chawla et al., 2013; Zhang et al., 2013). Thus, numerous studies have investigated the antioxidant capacity responses of higher plants to high salinity stress (Parida and Das, 2005; Ambede et al., 2012; Kurum et al., 2013; Hussain et al., 2014; AbdElgawad et al., 2016). Also, cyanobacteria were valuable models for studying the mechanisms behind tolerance to high salinity (Lu et al., 2006; Rao et al., 2007). Nevertheless, to our knowledge, the information regarding the antioxidant defense mechanisms of bacteria when exposed to high salinity stress is scarcely found. Therefore, in an attempt to innovate promising costless approaches for the synthesis of antioxidant postbiotics from bacteria, we investigated the influence of high salinity stress on the overall antioxidant capacity, as well as the enzymatic and non-enzymatic antioxidant mechanisms of four salt-tolerant strains isolated from marine environment that exhibited good growth capability and colonization under salt stress. To this end, several antioxidant metabolites and enzymes analyses were scaled down for semi–high-throughput analysis using a microplate reader. Assays were optimized to obtain a linear time and protein−concentration dependence. Moreover, the determination of GSH forms and phenolic and flavonoid profiles was performed by using the HPLC method. Based on the results of oxidative damage markers (MDA, lipoxygenase, and xanthine oxidase), it was found that salinity stress caused variable levels of oxidative damage to the selected four isolates. This was apparently clear in the two salt-tolerant strains from the genus *Planococcus* (ST3 and ST4), whereas the other two salt-tolerant strains of the genus *Bacillus* (ST1 and ST2) were less affected. In this context, elevations in MDA levels were reported by Zhang et al. (2013) in cyanobacteria, *Microcystis aeruginosa*, as a response to salinity stress. High salt was found to produce oxidative damage in various higher plants and tissues (Ashraf and Harris, 2004; Chawla et al., 2013). Through salt stress, the concentration of ROS rises in the tissues because of the disturbances in the electron transport chain and accumulation of photo-reducing power. This overabundance of electrochemical energy can be dispersed through the Mehler reaction, causing the formation of ROS including H_2_O_2_ (Asada, 1999), and damage of cellular membranes, which is reflected in higher MDA levels (Sharma et al., 2012; Chawla et al., 2013).

The results of oxidative stress damage of selected bacterial isolates in the present study correlated with the overall antioxidant capacity of these isolates, which was represented by FRAP, DPPH, and SOS. FRAP was significantly higher in *Planococcus* species (ST3) under both control and salinity stress conditions, followed by *Bacillus haikouensis* (ST2) when exposed to salinity stress. Also, ST2 and ST3 were higher in DPPH under control condition, which was reduced by exposure to salinity. Additionally, SOS was elevated in *Bacillus* strains (ST1 and ST2), while it was declined in *Planococcus* strains (ST3 and ST4) after exposure to salt stress. FRAP provides a direct estimation of the antioxidants or reductants present in a sample based on its ability to reduce the ferric (Fe^3+^)/(Fe^2+^) ferrous couple (Tanvir et al., 2015). DPPH is a stable nitrogen−centered radical that is commonly exploited to measure the free radical–scavenging activity of various samples; the higher the DPPH−scavenging activity of a certain sample, the higher the antioxidant activity of that sample. Velioglu et al. (1998) attributed that enhancement in the overall antioxidant activity to the phenolic compounds and flavonoids present in the extract of the selected fruits, vegetables, and grain products.

The changes in total antioxidant capacity are most likely explained by the changes in antioxidant metabolites such as flavonoids, phenols, and GSH (AbdElgawad et al., 2015). Correspondingly, we noticed that the present results of the oxidative stress damage of selected bacterial isolates under high salinity and consequently their total antioxidant capacity are attributed to non-enzymatic and enzymatic antioxidant mechanisms. Regarding the non-enzymatic antioxidant mechanism, we found that exposing selected bacterial isolates to salt stress resulted in significant elevations (*p* < 0.05) in the levels of polyphenols and flavonoids in almost all selected salt-tolerant strains, as well as in individual phenolic acids and flavonoids. Whereas GSH (reduced GSH, oxidized GSH, and total GSH) was increased in the two *Planococcus* species (ST3 and ST4) only, all forms of GSH were undetectable in the two *Bacillus* strains at both control and salinity stress conditions. Polyphenols act as potent antioxidants due to the hydrogen-donating capacity of their hydroxyl groups, in addition to their capability to donate electrons to stop the production of free radicals due to oxidative stress (Afroz et al., 2014), whereas flavonoids are the greatest class of polyphenols, which induce their action through scavenging or chelating processes (Schmitt-Schillig et al., 2005). In consistence with our results, Hichem et al. (2009) reported that salt-stressed maize plants produced high polyphenols. Besides, AbdElgawad et al. (2016) mentioned that roots and older leaves of maize responded to high salinity stress by increasing polyphenol contents and GSH levels. They attributed that finding to the augmented demand and metabolism of sulfur in the case of stress exposure for the biosynthesis of antioxidants such as GSH (Gill et al., 2013).

Interestingly, GSH production by the two species of genus *Planococcus* (ST3 and ST4) under control condition was apparently much higher than GSH levels from plant sources previously reported in earlier studies such as AbdElgawad et al. (2016), as they reported that total GSH levels did not exceed 0.3 nmol/g fresh weight of maize under either control or salinity stress, while here we reported that total GSH values produced by ST3 and ST4 accounted for 5.14 and 8.32 μmol/g cell weight, respectively, under control condition, which means several thousand folds that from higher plants. Also, the exposure of these two strains to salinity stress improved the GSH production to reach 6.71 and 13.68 μmol/g cell weight, respectively. Accordingly, growing these *Planococcus* species at high salt stress is a promising way for the production of the antioxidant GSH, which could be a good alternative to plant sources.

As regards the enzymatic antioxidant activities of selected bacterial isolates in response to salt stress, the results were also in correlation with the overall antioxidant capacity of the selected isolates. We found the enzymes that directly scavenge ROS, i.e., CAT, SOD, GPX, and POX, were significantly augmented in most selected isolates, particularly, CAT in *Planococcus* species (ST3 and ST4), SOD in ST1 and ST3, and GPX in ST1, ST3, and ST4, as well as POX in the two *Bacillus* strains (ST1 and ST2). Correspondingly, Zhang et al. (2013) stated a noteworthy increase in CAT and SOD activities in cyanobacteria, *M. aeruginosa*, at high salinity values (2.0 and 4.0 g NaCl L^–1^) as compared to control, whereas at low salinity values (0.5 and 1.0 g NaCl L^–1^) there was no significant difference than control. Also, Li et al. (2004) reported that ROS in *Chlorella ellipsoidea* algal cells was excessively generated, and SOD activity was significantly improved under high Cd^2+^ stress. Numerous antioxidant enzymes, such as CAT and SOD, are implicated in the detoxification of ROS and the prevention of damage due to high salinity stress (Cavalcanti et al., 2007; Sekmen et al., 2007).

A similar scenario was observed in *Planococcus* species (ST3 and ST4) in the case of enzymes that reduce the antioxidant metabolites, namely, GR, Grx, and TRD; they were elevated by high salt exposure. On the other hand, these enzyme activities were negligible in the two *Bacillus* strains (ST1 and ST2) under both control and salt stress conditions. Regarding the detoxification enzyme (GST), in consistence with GSH production results, it was undetectable in the two *Bacillus* strains (ST1 and ST2) under both control and salinity stress conditions, although it was significantly elevated in the two *Planococcus* species (ST3 and ST4) by exposing to salinity stress.

## Conclusion

Exposing the selected four salt-tolerant bacterial strains to salinity stress resulted in variable degrees of oxidative damage; consequentially, these microorganisms exploited their antioxidant defense system to overcome salinity-induced cellular damage. Strain-specific response to salt stress was noticeable. *Planococcus* species (ST3 and ST4) showed increased GSH cycle metabolites and enzyme activities, polyphenols, flavonoids, total antioxidant capacity, CAT, and SOD. On the other hand, the improvement of antioxidant production in salt-stressed *Bacillus* species (ST1 and ST2) was restricted to the polyphenols, flavonoids, POX, Grx, and/or SOD enzymes. GSH production by these species of the genus *Planococcus* (ST3 and ST4) under control condition apparently was much higher than GSH levels from plant sources, as well as the exposure of these strains to salinity stress duplicated GSH production. Accordingly, growing these *Planococcus* species at high salt stress is a promising way for the production of the antioxidant GSH, as a good alternative to plant sources. Thus, we herein report for the first time that salinity stress promotes the production of various antioxidant metabolites and enzymes from these selected strains of *Bacillus* and *Planococcus*, which can be utilized as postbiotics for potential application as functional additives to foods and pharmaceuticals to enhance food stability and to promote human health.

## Data Availability Statement

The raw data supporting the conclusions of this article will be made available by the authors, without undue reservation.

## Author Contributions

AH, WH, and HA conceived the study. WH, DA, and SA isolated and identified the bacterial strains. AH and HA conducted salinity stress exposure, assessed the antioxidant capacity, analyzed the results, and performed the statistics and illustrations. AM and HA determined the phenolic and flavonoid profiles. AH, WH, HA, and GB wrote the manuscript. WH, GB, DA, and SA contributed to project funding. All authors reviewed the submitted version.

## Conflict of Interest

The authors declare that the research was conducted in the absence of any commercial or financial relationships that could be construed as a potential conflict of interest.
