# Supplementary material for: Salinity Stress Enhances the Antioxidant Capacity of Bacillus and Planococcus Species Isolated From Saline Lake Environment
Source: Front Microbiol. 2020 Sep 14;11:561816. doi: 10.3389/fmicb.2020.561816 (PMC7521018; doi:10.3389/fmicb.2020.561816)
Supplement: Supplementary file 1 [file Data_Sheet_1.docx]

**Supplementary Material**

**Salinity Stress Enhances the Antioxidant Capacity of *Bacillus* and *Planococcus* Species Isolated from Saline Lake Environment**

**Abdelrahim H. A. Hassan^1*^, Dalal Hussien M. Alkhalifah^2^, Sulaiman A. Al Yousef^3^, Gerrit T.S. Beemster^4^, Ahmed S. M. Mousa^6^,** **Wael N. Hozzein^5,6^, Hamada AbdElgawad^6^**

^1^ Department of Food Hygiene and Control, Faculty of Veterinary Medicine, Beni-Suef University, Beni-Suef 62511, Egypt

^2^ Biology Department, College of Science, Princess Nourah Bint Abdulrahman University, Riyadh, Saudi Arabia

^3^ Clinical Laboratories Sciences Department, College of Applied Medical Science, Hafr Al Batin University, Hafr Al Batin-31991, Saudi Arabia

^4^ Integrated Molecular Plant Physiology Research, Department of Biology, University of Antwerp, Antwerp, Belgium

^5^ Bioproducts Research Chair, Zoology Department, College of Science, King Saud University, Riyadh, 11451, Saudi Arabia

^6^ Botany and Microbiology Department, Faculty of Science, Beni‒Suef University, Beni‒Suef, 62521, Egypt

**^*^ Correspondence:**

Abdelrahim Hassan

[abdelrahim@vet.bsu.edu.eg](mailto:abdelrahim@vet.bsu.edu.eg)

**Table S1: The optical density (absorbance OD_600 nm_) values of different bacterial cultures after 24 h of incubation.**

| **Collection time** | **ST1** | | **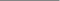ST2** | | **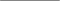ST3** | | **ST4** | |
| --- | --- | --- | --- | --- | --- | --- | --- | --- |
|  | **Control** | **Salinity stress**  **(20% NaCl)** | **Control** | **Salinity stress**  **(20% NaCl)** | **Control** | **Salinity stress**  **(20% NaCl)** | **Control** | **Salinity stress**  **(20% NaCl)** |
| **24 h** | 2.54± 0.08^ab^ | 2.82± 0.12^a^ | 2.78± 0.4^a^ | 3.05± 0.31^a^ | 0.81± 0.09^cd^ | 0.98± 0.15^c^ | 1.05± 0.12^c^ | 1.34± 0.07^c^ |

Different small letters (a, b, c) superscripts indicate significant differences between means of OD at 24h of incubation.


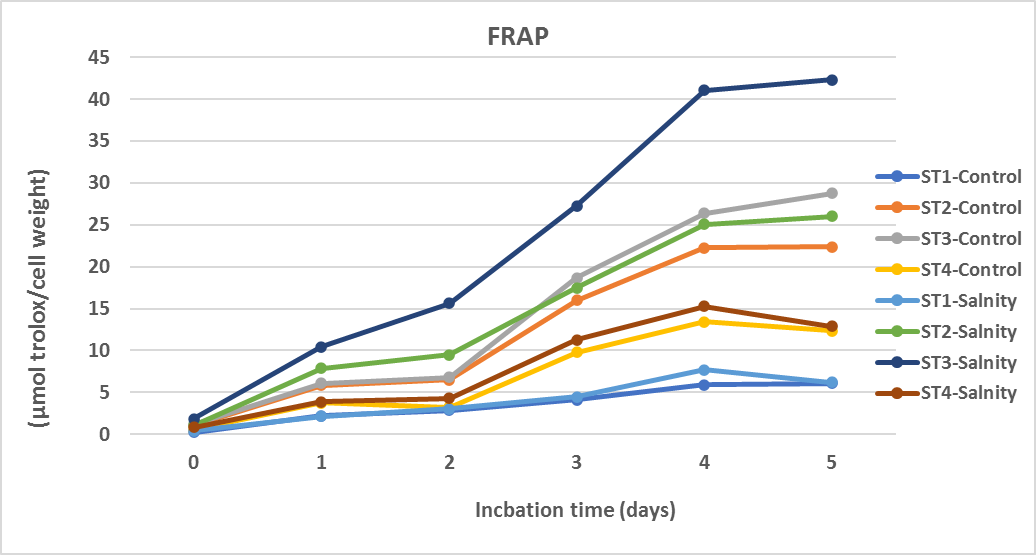


**Figure S1. The preliminary results of the total antioxidant capacity, ferric reducing/antioxidant power (FRAP) obtained by the four selected salt tolerant (ST) cultures at control or salinity stress.**
